# Representational formats of human memory traces

**DOI:** 10.1007/s00429-023-02636-9

**Published:** 2023-04-06

**Authors:** Rebekka Heinen, Anne Bierbrauer, Oliver T. Wolf, Nikolai Axmacher

**Affiliations:** 1 Department of Neuropsychology, Institute of Cognitive Neuroscience, Faculty of Psychology, Ruhr University Bochum, Universitätsstraße 150, 44801 Bochum, Germany; 2 Institute for Systems Neuroscience, Medical Center Hamburg-Eppendorf, Martinistraße 52, 20251 Hamburg, Germany; 3 Department of Cognitive Psychology, Institute of Cognitive Neuroscience, Faculty of Psychology, Ruhr University Bochum, Universitätsstraße 150, 44801 Bochum, Germany

**Keywords:** Memory, Neural representations, Representational similarity analysis, Representational formats, Deep neural networks

## Abstract

Neural representations are internal brain states that constitute the brain’s model of the external world or some of its features. In the presence of sensory input, a representation may reflect various properties of this input. When perceptual information is no longer available, the brain can still activate representations of previously experienced episodes due to the formation of memory traces. In this review, we aim at characterizing the nature of neural memory representations and how they can be assessed with cognitive neuroscience methods, mainly focusing on neuroimaging. We discuss how multivariate analysis techniques such as representational similarity analysis (RSA) and deep neural networks (DNNs) can be leveraged to gain insights into the structure of neural representations and their different representational formats. We provide several examples of recent studies which demonstrate that we are able to not only measure memory representations using RSA but are also able to investigate their multiple formats using DNNs. We demonstrate that in addition to slow generalization during consolidation, memory representations are subject to semantization already during short-term memory, by revealing a shift from visual to semantic format. In addition to perceptual and conceptual formats, we describe the impact of affective evaluations as an additional dimension of episodic memories. Overall, these studies illustrate how the analysis of neural representations may help us gain a deeper understanding of the nature of human memory.

## Introduction: why should we assume representations?

When we think back to what we did yesterday, we are usually able to literally *picture* how a specific episode looked like, and perhaps also how it sounded, smelled, and felt. This ability to form a mental image or internal representation plays a crucial role for both re-experiencing the past and making plans for the future (Schacter and Addis 2007; Bonnici et al. 2012; Cheng et al. 2016; Brown et al. 2016). How is the sensory information about this episode transformed into a long-lasting neural memory trace? Will different aspects such as visual and abstract information be stored differently in memory? How can we measure the representational format of memories?

First of all: What is a representation? Described as early as 1904 by Richard Semon (e.g., Schacter 2001), most cognitive neuroscientists nowadays believe that mental representations of past and future episodes rely on a neural substrate that we can localize in the brain—on the “neural representation” of the represented episode (deCharms and Zador 2000; Shea 2018)—this notion has not always been accepted. Beginning with the “cognitive revolution” in the 1960s, cognitivism replaced behaviorism, a scientific movement trying to explain behavior not only without introspection, but also without assuming mental representations (Watson 1913; Skinner 1953; Egan 2014; Shea 2018; Newen and Vosgerau 2020). In contrast to behaviorism, cognitivists emphasized the importance of intentional states and mental contents for understanding cognitive functioning. According to this representational view, a mental representation consists of (1) a vehicle, i.e., a physical entity such as a population of neurons that is able to represent information, and (2) a content, i.e., the information about the outside world or about internal states that is carried by the vehicle (Fodor 2008; Roskies 2021). In addition—and critical for our review—this content can have (3) different representational formats: A given experience can be either represented conceptually or non-conceptually (Boghossian 1995). While conceptual representational formats are composed of semantic thoughts, non-conceptual formats rely on sensory aspects of an experience. Arguably, most “real-life” representations consist of both representational formats. For example, the representation of a visit to the ocean (content) comprises the fact that one was at a certain beach at a certain time (conceptual representational formats) and the feeling of sand beneath one's feet, the color of the water, and the heat of the sun (non-conceptual representational formats). The brain states carrying both types of information constitute the vehicles of mental representations. In this review, we will focus on these two types of formats—perceptual and conceptual.

The representational theory of the mind assumes that cognitive functioning consists of the formation and the transformation of mental representations. It will thus be important to develop methods to measure these representations and assessing their vehicle in the brain has become a core aim of contemporary cognitive neuroscience.

## A case for internal representations

“A neural representation is a pattern of neural activity that stands for some environmental feature in the internal workings of the brain” (Vilarroya 2017, p. 4) and focuses on particular features in the world—i.e., neural representations have a representational content and involve a particular representational format (deCharms and Zador 2000). At early steps of sensory processing, neural representations involve representational formats that are more strongly correlated with external input than at later processing stages. For example, Hubel and Wiesel (1959) studied how the early visual cortex responds to bars at different angular directions. The striate cortex and other cortices at the beginning of the sensory processing hierarchy exhibit pronounced topographic organization, such that the patterns of activity are isomorphic with the external world (Poldrack 2021). At later processing steps, neural representations are less strongly driven by sensory inputs and more strongly shaped by cognitive operations. A famous example of such a representation occurs in an experiment that Tolman described in his book “Cognitive maps in rats and men” (1948): A rodent explores a maze and may find rewards when choosing the correct path. After some time, the reward path is blocked, and the rodent is offered several different alternative paths. Tolman could demonstrate that rodents took the shortest alternative path. This is indicative of an internal representation—in this case of relative spatial locations—that is referred to as “cognitive map”, as the behavior of the rodent cannot be solely explained by stimulus–response learning based on stimulus-outcome associations.

## How can we measure and analyze neural representations?

Out of many ideas and possibilities how stimulus information is represented in neural structures, three prominent theories evolved which differ regarding the neural features containing representations. On the level of single neurons, the ‘rate coding’ hypothesis claims that the mean firing rate of each neuron carries information about stimuli (Adrian 1928; DeCharms and Zador 2000). The ‘temporal coding’ hypothesis posits that in addition to the mean firing rate the precise timing of spikes is crucial (DeCharms and Zador 2000; Gerstner and Kistler 2002; Gollisch and Meister 2008). We consider these coding schemes on the single unit level as “sparse” since they focus on coding by one or a few neurons (Axmacher et al. 2008; Reddy and Kanwisher 2006). In addition, the activity of large populations of neurons also carries information (Deadwyler and Hampson 1997; DeCharms and Zador 2000; Georgopoulos et al. 1986; Hebb 1949). This scheme of ‘population coding’ would be consistent with a large number of broadly tuned neurons that code for a given stimulus (Reddy and Kanwisher 2006).

At the population level, neural representations can be measured by decoding approaches which can be applied to various kinds of non-invasive data in human participants (most importantly, functional magnetic resonance imaging, fMRI, or electroencephalography, EEG). In contrast to univariate analysis techniques which reflect overall activity changes a commonly used way to assess neural representations in cognitive neuroscience is multivariate pattern analysis (MVPA). With the advent of MVPA it has become possible to extract representational contents and formats from distributed patterns of neural activity, e.g., voxel activity values in fMRI data or power values at various frequency bands, time points, and channels in EEG data (Naselaris et al. 2011; Hebart and Baker 2018; Kunz et al. 2018; Roskies 2021).

When two stimuli elicit similar overall activity levels and their informational content is reflected by the pattern of voxel activations instead, it may be impossible to find univariate activation differences. Therefore, MVPA aims at decoding the information that the patterns of activity carry about external stimuli (Haynes and Rees 2006; Kriegeskorte et al. 2008a; Mur et al. 2009; Haxby et al. 2014; Kragel et al. 2018). Even when brain regions are relevant for processing a large number of different stimuli, it thus becomes possible to differentiate neural representations of two stimuli based on their activation pattern (Mur et al. 2009; Raizada et al. 2010), which may reflect a neural population code (Kamitani and Tong 2005; Watrous et al. 2015; Kriegeskorte and Diedrichsen 2019).

The underlying assumption of MVPA is that neural representations can be characterized via high-dimensional state-spaces whose dimensions correspond to stimulus attributes, and that each individual representation corresponds to one point in this space (Haxby et al. 2014). The two most commonly used MVPA methods are pattern classification (Pereira et al. 2009) and representational similarity analysis (RSA; Kriegeskorte et al. 2008a; Kriegeskorte and Diedrichsen 2019).

RSA allows researchers to characterize the geometry of a representational space that can be based on various stimulus features (Kriegeskorte and Kievit 2013; Haxby et al. 2014; Kriegeskorte and Wei 2021; Roskies 2021). Importantly, RSA abstracts from the specific type of data that is investigated (e.g., fMRI or EEG) and consists of a matrix of similarities which quantifies the (dis)similarity between neural representations (Haxby et al. 2014). Hence, it becomes possible to analyze second-order similarities—i.e., the correspondence between two separate similarity matrices (RDMs)—of (1) neural representations measured in different brain regions, species, or modalities, (2) neural activity and behavioral outcomes, or (3) neural activity and computational models (Kriegeskorte et al. 2008a; Kriegeskorte and Kievit 2013; Haxby et al. 2014; Roskies 2021). In other words, RSA allows for an analysis of any kind of data pattern irrespective of the data format.

Implementing RSA requires the coding of neural activity as vectors, separately for the experimental conditions (e.g., for stimuli in an experiment). Afterwards, the representational distances between these vectors are calculated. The similarity or distance measures that are most often used are Pearson or Spearman correlations, or Euclidean or Mahalanobis distance. Higher similarity corresponds to lower representational distance and vice versa. The result is a representational dissimilarity matrix (RDM; Kriegeskorte et al. 2008a), i.e., a matrix that reflects the similarities or distances between every stimulus (or more generally, condition) with every other stimulus, resulting in a *n*x*n* matrix (e.g., stimulus x stimulus, condition x condition). Approaches like multidimensional scaling allow for a mapping of this high-dimensional representational space in lower-dimensional spaces (often 2D or 3D) in order to facilitate interpretation.

We now can extract information from the RDMs to characterize the underlying neural representations. First, self-similarity, sometimes also called representational fidelity or reliability (see Xue 2018 for review), refers to the similarity of brain patterns when the same stimulus is presented twice. Although self-similarity most commonly refers to the similarity of a stimulus compared to others (i.e., to non-self similarity), some studies use this term to denote the similarity between repetitions of the same stimulus (i.e., Xue et al. 2010). Here, we use this term to refer to the first case (within vs. between similarity). This tells us how faithful a neural representation reflects a given stimulus. Second, RDMs allow us to investigate the relationships between different stimuli, i.e., between-item similarity. This between-item similarity may reflect the features of a stimulus that are represented by a given brain region—i.e., two stimuli with similar low-level visual features, such as spatial frequencies or gratings, have similar representations in early visual cortices, while conceptual similarities lead to similar representations in association cortices (Kriegeskorte et al. 2008a, b). Based on these differences, RSA allows unraveling the representational format of neural representations. This method is highly flexible since many different features or conditions can be investigated in one experiment. One possible application is the investigation of human episodic memory, which we will describe next.

## How do neural representations relate to memory?

An episodic memory can be conceived of as an internal representation of a previous experience (Goldman-Rakic 1995; Brewer et al. 1998; Cheng et al. 2016; Vilarroya 2017). At the neural level, it is widely assumed that memory representations are stored in memory traces or engrams—a term coined by Richard Semon in order to refer to learning-induced alterations of brain (micro-)structure (Semon 1904, 1909).

According to Semon, engrams are biological states that are objectively observable, which means that in principle, we can locate and manipulate them (Semon 1904, 1909; Josselyn et al. 2015; Kunz et al. 2018). Second, they represent specific memory contents and thus, when activated, lead to expression of this memory content (i.e., behaviorally measurable memory retrieval) (Liu et al. 2014a, b; Kunz et al. 2018). Moreover, they may be distributed within and across brain areas, an aspect that had been suggested by Lashley (1950) and was empirically supported for encoding by Haxby et al. (2001) and for retrieval by Brodt et al. (2018) more than 50 years later. Each engram corresponds specifically and uniquely to one particular memory, and this relationship is stable such that a given engram, when activated, should always elicit the same memory (Han et al. 2009; Liu et al. 2012, 2014a, b; Kunz et al. 2018). However, engrams or memory traces may also be transformed by various factors, such as time, memory consolidation, and novel learning (Dudai et al. 2015).

The formation of episodic memories requires the representation of the episode in a lasting memory trace (Xue 2018). In humans, various characteristics of memory representations have been associated with episodic memory performance: First, high amounts of self-similarity—i.e., of the memory representation of a particular content—predict subsequent memory (Xue et al. 2010; Visser et al. 2013). This result was found across various brain regions involving frontoparietal areas, the posterior cingulate cortex and sensory regions that are involved in processing the respective stimuli (Xue 2018). Self-similarity of memory representations may either refer to situations when a particular stimulus is encoded multiple times (encoding-encoding-similarity) or when encoding and retrieval of the same stimulus are compared (encoding-retrieval-similarity), and both measures predict memory accuracy (Xue et al. 2010; Xue 2018; Ten Oever et al. 2021). Thus, higher similarity between memory representations of the same stimulus seems to support (recognition) memory.

Interestingly, between-item similarity has been associated with memory performance as well, although not necessarily in a positive manner: Indeed, different theoretical frameworks and empirical results predict a memory advantage either for more distinct or for more similar memory representations of different items. Some studies found that stronger discrimination between different items (i.e., higher distinctiveness) supports memory (LaRocque et al. 2013; Xue 2018). The distinctiveness hypothesis is based on the idea that distinctiveness reduces possible interference with other, similar stimuli and thereby supports memory (Kılıç et al. 2017). The idea of distinctiveness is closely related to pattern separation in the hippocampus, a process by which similar memories are stored as distinct, non-overlapping representations (Bakker et al. 2008; Yassa and Stark 2011). An fMRI study confirmed that higher pattern distinctiveness in the hippocampus is indeed associated with better memory performance (LaRocque et al. 2013). In contrast, in perirhinal and parahippocampal cortex as well as in the amygdala, higher between-item similarity of neural representations benefits memory encoding, possibly because they are integrated into one unique episode that is distinct from other episodes (Visser et al. 2011, 2013; LaRocque et al. 2013; Bierbrauer et al. 2021). While these studies point to better memory performance with higher distinctiveness, there is also evidence that global pattern similarity—i.e., the similarity between different exemplars of the same concept—may support memory (Davis et al. 2014), even causing false alarms for new exemplars of the same concept (Wing et al. 2020).

These results support the idea that memory representations have multiple representational formats, whose representational ‘geometries’ (generalized or distinct) may exert different influences on memory encoding. In this review, we define visual/perceptual representational formats as reflecting visual stimulus features (e.g., their colors, textures, or shapes). Conversely, we define conceptual/semantic formats as reflecting semantic stimulus features including category information. How can we quantify the representational formats and measure the degree to which a stimulus might be represented in a visual or a conceptual format?

## Using deep neural networks as models of representational formats

In recent years, the field of artificial intelligence has revolutionized our lives, with artificial neural network models (ANN) achieving near human-like performance in areas such as language translation (Popel et al. 2020) and car driving (Gupta et al. 2021) and even out-performing humans in various complex games such as chess (McGrath et al. 2022), Go (Silver et al. 2017), Starcraft (Vinyals et al. 2019) or Stratego (Perolat et al. 2022). If ANN models are able to perform on a human level, can we also utilize them to better understand our own brain processes?

**Fig. 1:**
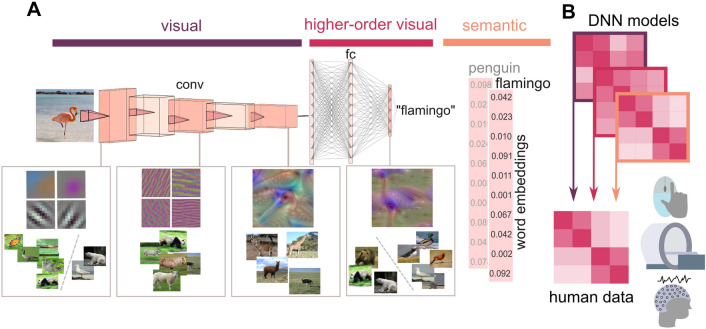
Linking representational formats in DNNs and in semantic models to representations in the human brain. **A** Different representational formats in convolutional deep neural networks (cDNNs) and deep natural language processing models (dNLPs). In cDNNs, images are processed with a gradient in complexity, comparable to the human ventral visual pathway. As in the brain, basic visual information is processed in the first layer (whose receptive field properties roughly match those of V1) and is then passed through the convolutional layers of the network, which process increasingly complex information. After the last convolutional layer, the connections change, as now each neuron is connected to each neuron in subsequent layers (fully-connected). The network then chooses the most active (i.e., most likely) label in the highest layer. Early layers of the cDNN process edges, colors, and textures, such that e.g., animals of different categories (species) are sorted together e.g., based on their color. DNN neurons in middle layers have more complex receptive fields, processing object features such as the beak of the flamingo or their long necks and legs. Late layers respond to a visual prototype of the flamingo and show distinct representational similarity patterns (e.g., all bears are sorted together, even if they differ in their low-level visual features). While convolutional layers process lower-level sensory information, fully-connected layers process higher-order visual features including object classes. In addition to the cDNN, one can use dNLP models to quantify the representation of semantic information, based on embedding vectors of words or sentences. **B** The neural activations corresponding to all pairs of stimuli can be used to generate RDMs for all layers of the cDNN and the semantic dNLP, which can then be correlated to RDMs from brain or behavioral data generated using the same stimuli

To gain insight into the transformation of visual features into conceptual representations, convolutional Deep Neural Networks (cDNNs) from object recognition (Fig. 1A) have become models of choice. These models process image input through several convolutional layers, which are connected sparsely, up to fully-connected layers that assign a label to contents of the image. Strikingly, recent multivariate studies have found the same visual hierarchy and gradient in feature complexity in cDNNs trained on object recognition as observed in the brain (Leeds et al. 2013; Khaligh-Razavi and Kriegeskorte 2014; Güçlü and van Gerven 2015; Yamins and DiCarlo 2016; Cichy et al. 2016; Wen et al. 2018). These results were obtained across various data modalities, ranging from fMRI (Güçlü and van Gerven 2015; Allen et al. 2022) via magnetoencephalography (MEG; Clarke et al. 2018) and scalp EEG (Graumann et al. 2022) to oscillations in intracranial EEG (Kuzovkin et al. 2018) and monkey single-unit data (Cadieu et al 2014) and even behavioral outcomes, such as similarity judgements (Mur et al. 2013; Davis et al. 2021).

These findings demonstrate that the internal representations on multiple levels of complexity formed by cDNNs are closely linked to the features that are processed along the ventral visual stream (VVS) (Fig. 1A). Processing of visual information along the VVS reveals a hierarchy from basic visual features to higher-order visual and semantic category features (Cowell et al. 2010; DiCarlo et al. 2012; Kravitz et al. 2013). The visual cortex processes low-level visual properties, such as colors, shapes, and textures, and neurons in these areas have small receptive field sizes that lead to pronounced retinotopic specialization (Hubel and Wiesel 1962). As the signal progresses through the VVS to more anterior regions, such as the inferior temporal cortex (IT cortex; Kriegeskorte et al. 2008b), the fusiform gyrus (Clarke et al. 2011) and the lateral occipital cortex (LOC; Tyler et al. 2013), feature complexity and receptive field size increase, leading to higher-order representational formats involving object parts and domain-level semantic features (Clarke et al. 2013; Clarke 2015).

In cDNNs from object recognition, starting with low-level features such as edges and colors in the first convolutional layer, complexity increases to textures, object parts and finally, object categories in the last network layer. While early cDNN layers show similar activation patterns for images with shared visual features such as similar colors and textures (e.g., orange color of a pumpkin and of a basketball), independent of the conceptual similarity of stimuli, category-specific features explain similarities in later layers (e.g., wings, feathers and a beak for birds). Similar representational transformations have been found along the VVS (Mur et al. 2013; Hebart et al. 2020), revealing that cDNN models from computer vision can accurately reflect neural representations during object recognition. Surprisingly, in contrast to this functional overlap, the most prominently used network “AlexNet” (Krizhevsky et al. 2017) contains one of the simplest architectures. Yet, AlexNet and other, shallower cDNN models such as CorNet (Kubilius et al. 2018, 2019) are models that match neural representations relatively well (Nonaka et al. 2021) and show high classification performance in object recognition. On the other hand, it has been demonstrated that recurrency in DNN architectures may further improve the match to neural representations during object recognition (Kubilius et al. 2019; Kietzmann et al. 2019b; van Bergen and Kriegeskorte 2020), suggesting that recurrent DNNs should be increasingly used in the future to study neural representations.

Neural representations can be mapped onto DNN feature spaces via RSA, using the fact that RSA reflects representational geometry independent of data modality (Fig. 1B). Treating DNN feature activations as patterns allows one to compute similarities between all pairs of stimuli in each layer of the DNN or model, resulting in one RDM per DNN layer/model. Subsequently, DNN RDMs and neural RDMs can be correlated and compared for their similarity structures (Mur et al. 2013; McClure and Kriegeskorte 2016). Several visualization techniques such as multi-dimensional scaling (MDS; Lin et al. 2019; Fig. 1A), class activation maps (Zhou et al. 2015) or similarities from RDMs (Kriegeskorte and Golan 2019) provide information on the representational formats that are processed in individual DNN layers or models. Linking DNN representations to neural representations thus allows one to examine properties of neural representations (e.g., in terms of brain regions, oscillation frequencies, or latencies) that reflect different representational formats and are for example responsible for the shift from perceptual to conceptual formats.

Many studies mentioned above employed cDNN models from image classification challenges to assess representational formats during neural processing. However, these cDNN models are limited to visual and higher-order visual representations, while category abstraction and many memory functions rely on conceptual representations (Clarke 2019). More specifically, even though perceptual features may allow for the derivation of conceptual representations in a feed-forward way (Clarke et al. 2018), this process can be facilitated by top-down semantic knowledge (Taylor et al. 2012; van Kesteren et al. 2013), emphasizing the need for models that involve semantic processing. In fact, research on language processing even provided evidence for multiple levels of semantic features, as indicated by faster performance for general domain features compared to exemplar-specific features (Randall et al. 2004; Macé et al. 2009; Devereux et al. 2018). Thus, instead of focusing on one single cDNN model, Clarke (2019) proposed the additional use of deep learning models from natural language processing (deep Natural Language Processing models; dNLP). Previous research showed that these models can accurately reflect conceptual representations during object recognition in the VVS (Devereux et al. 2018) and even during more abstract tasks such as those involving narrative content (Lee and Chen 2022). DNLP models are trained on text input (e.g., wiki pages, books, user reviews) rather than images. These corpus-based models, such as BERT (Devlin et al. 2018), the Google Sentence encoder (Cer et al. 2018), Infersent (Conneau et al. 2017) or GPT-3 (Brown et al. 2020) assign a word embedding vector to each word or sentence based on co-occurrences of these concepts, and these vectors can then be used to study semantic similarities.

Taken together, although cDNN models are only very rough approximations to the neural processes and connectivity within the VVS, the findings reviewed above demonstrate that representations in DNN layers are relatively accurate models of the neural representations at different levels of abstraction, which makes them specifically interesting to study neural properties of and changes in representational format (Marblestone et al. 2016; Kietzmann et al. 2019a; Richards et al. 2019; Storrs and Kriegeskorte 2019; Saxe et al. 2021). Surprisingly, only few studies thus far employed DNNs to study representational formats of memory representations. Davis et al. (2021) were among the first to apply visual and semantic DNN model features to investigate the effects of representational formats during encoding on subsequent memory. Participants first viewed images of natural objects that they had to name and were then tested in two retrieval tasks. During retrieval, the authors separately made either perceptual or conceptual formats task-relevant by either displaying old and new images (perceptual) or the label of the concepts (conceptual). Using fMRI, the authors could show that matching of encoding representations to RDMs from either a visual cDNN or semantic models (taxonomy/encyclopedic) predicted memory performance in both retrieval conditions. Conceptual and perceptual formats recruited different brain areas though, namely the anterior VVS and the early visual cortex, respectively. Interestingly, although the two representational formats were linked to different brain areas depending on the retrieval task (perceptual/conceptual), the performance in both tasks benefited from matching with the respective other format during retrieval as well, suggesting that perceptual memory benefits from top-down information, while bottom-up visual information facilitates conceptual memory.

## The role of representational formats for understanding the dynamics of memory representations

Several findings of item-specific memory representations concern frontoparietal and midline regions (e.g., Baldassano et al. 2017; Fernandino et al. 2022; Huth et al. 2012; Lee and Kuhl 2016). Since these areas do not reflect sensory processing, it is currently not clear why they exhibit pronounced stimulus specificity. In the future, DNNs trained on more complex objective functions than stimulus categorization may account for the formats in these areas. However, studies using RSA identified an important role of the VVS in transforming visual into conceptual representations (DiCarlo et al. 2012; Kravitz et al. 2013; Martin et al. 2018). This transformation from perceptual to conceptual representations during perception (Kriegeskorte and Kievit 2013) may also give rise to different representational formats of memory traces, which may rely predominantly on either perceptual or semantic representational formats as well. Since both visual and semantic formats play a role during object recognition, the question arises whether memory traces during the different stages of memory processing—encoding, short-term memory maintenance, consolidation, and retrieval—would reveal such formats as well. Already at early visual processing steps, top-down knowledge plays an important role. Typically, we do not encounter objects without any prior information on their use and behavioral importance but using conceptual representations that are stored in long-term memory (Tulving and Watkins 1975; Xue 2018). At the same time, neural representations are not stable but subject to transformation processes (Xue 2022). According to the neural-psychological-representation-correspondence (NPRC) by Gilboa and Moscovitch (2021) memory traces can occur in different forms, a given episode can be represented in an event-specific visual format, while at the same time containing information about schemas and semantic information from prior knowledge. In addition, these representational formats may dynamically change due to various factors such as time after encoding, task context, goals, or prior knowledge, resulting in transformations between formats. How are these representations formed and especially, how are they transformed?

One candidate framework on the transformation of visual information into long-term memory is based on the concept of semantization or gist-abstraction (Konkle et al. 2010; Winocur and Moscovitch 2011; Linde-Domingo et al. 2019; Lifanov et al. 2021). During semantization, sensory information is integrated into long-term semantic knowledge through representational transformations (Paller and Wagner 2002; Xue 2018; Favila et al. 2020). According to this framework, conceptual features of a sensory input are selectively strengthened, while detailed sensory information is reduced, facilitating the integration of novel experiences with prior semantic knowledge. In line with this theory, studies found better memory performance for conceptual features as compared to low-level/perceptual features (Bainbridge et al. 2017; Bainbridge 2019; Linde-Domingo et al. 2019). Memory was also improved for stimuli that could easily be linked with pre-existing schemas as compared to those that did not match a schema (van Kesteren et al. 2013), and reaction times were faster for conceptual compared to perceptual features during recall (Lifanov et al. 2021). Thus, semantization can be defined as a transformation from detail-rich to compressed gist-like representations, suggesting a change in representational format. In addition, one would expect a transformation of memory traces such that they become more similar for stimuli that share the same prototypical conceptual features (e.g., beak and wings of birds) and less similar for stimuli with similar visual details (e.g., red parrot and red tomato).

In this case, semantization may actually lead to an increase of false alarms to semantically similar lures or to novel exemplars of previously presented concepts. Indeed, Naspi et al. (2021a) found more errors for lures consisting of prototypical exemplars of a given category, indicating that enhanced gist-abstraction during encoding or consolidation can lead to increased false alarms at recognition. At the same time, false alarms also increased for lures with high visual similarity to originally encoded images, suggesting that not all unique visual information is lost after encoding. Delhaye and Bastin (2021), who focused on the impact of visual or semantic processing during encoding, found semantization to be independent of encoding type format. Interestingly, Naspi et al. (2021b) could show that both visual and semantic formats in VVS contributed to successful memory encoding, but categorical information in regions anterior to the VVS predicted later forgetting. These studies demonstrate that there is no rigid transformation from visual to semantic formats during encoding but an interplay between different formats at different steps of memory processing.

A substantial body of evidence has shown off-line replay of memory representations during sleep (Frankland and Bontempi 2005; Deuker et al. 2013; Dudai et al. 2015). Integration of novel experiences into prior knowledge is assumed to be caused by strengthening of those features that are shared across encoded contents (Káli and Dayan 2004; Lewis and Durrant 2011; Himmer et al. 2019). These results are in line with the idea that replay facilitates generalization processes (Liu et al. 2019). While sleep contributes to integration by enhancing memory for shared features of newly encoded content, there is also evidence for sleep to prevent loss of unique feature representations (Schapiro et al. 2017). This might indicate that not only conceptual but multiple representational formats, including perceptual details, are strengthened due to off-line replay during sleep, which in turn might slow down the supposed loss of visual detail over time.

Perceptual details might be subject to faster forgetting in order to promote an integration of conceptual or super-ordinate categorical features into memory, considering that different representational formats of a memory trace may be forgotten independently (Brady et al. 2013). In line with supposed gist abstraction and loss of visual detail due to semantization, Lifanov et al. (2021) found that a perceptual-conceptual gap (e.g., a shift from faster reaction times for perceptual features to faster reaction times for conceptual features) increased over time, suggesting faster forgetting of visual details while conceptual features were integrated into long-term memory (LTM). During retrieval, conceptual features were activated prior to visual detail when no visual input was present (Linde-Domingo et al. 2019; Davis et al. 2021) and were found to be involved during memory retrieval of both perceptual and conceptual representational formats (Davis et al. 2021; see above). While these results could lead to the wrong conclusion of a complete loss of visual detail over time, Ferreira et al. (2019) found higher neural similarities between category-related but also between episode-unique information, demonstrating that conceptualization during semantization does not necessarily come at the cost of visual detail.

Overall, these findings deliver further evidence for a dynamic transformation of representational formats (Paller and Wagner 2002; Xue 2018, 2022; Favila et al. 2020; Liu et al. 2020, 2021). Yet, the question remains whether consolidation induces transformations of memory traces from one format to another (i.e., from perceptual to conceptual, losing all perceptual detail) or whether memory traces consist of multiple formats with only their accessibility changing across time, and depending on encoding tasks and/or retrieval cues. While this can be investigated by analyses of encoding-retrieval similarity (i.e., Ten Oever et al. 2021), DNNs could be used to further investigate the underlying representational formats and to address the question whether these formats are subject to transformation or continue to coexist. In the next section of this review, we will take a closer look at how DNNs may be used to investigate such changes in the representational format of memory traces during the earliest possible stage when semantization might occur, i.e., directly after the offset of a stimulus, and during subsequent processing stages.

## Beyond recognition—using DNNs to investigate early stages of semantization

As described above, previous research has demonstrated that cDNN features can be used to study representational formats during object recognition (Güçlü and van Gerven 2015; Cichy et al. 2016; Kuzovkin et al. 2018; Clarke et al. 2018). However, these studies did not assess the question of how these visual inputs were transformed during the consecutive stages of long-term memory encoding, memory consolidation, and long-term memory retrieval. Investigating representational formats during initial stages of memory formation, we could test whether the supposedly slow process of gist-abstraction (O’Reilly et al. 2014) unfolding during systems consolidation might happen more rapidly and already in earlier post-encoding stages.

**Fig. 2:**
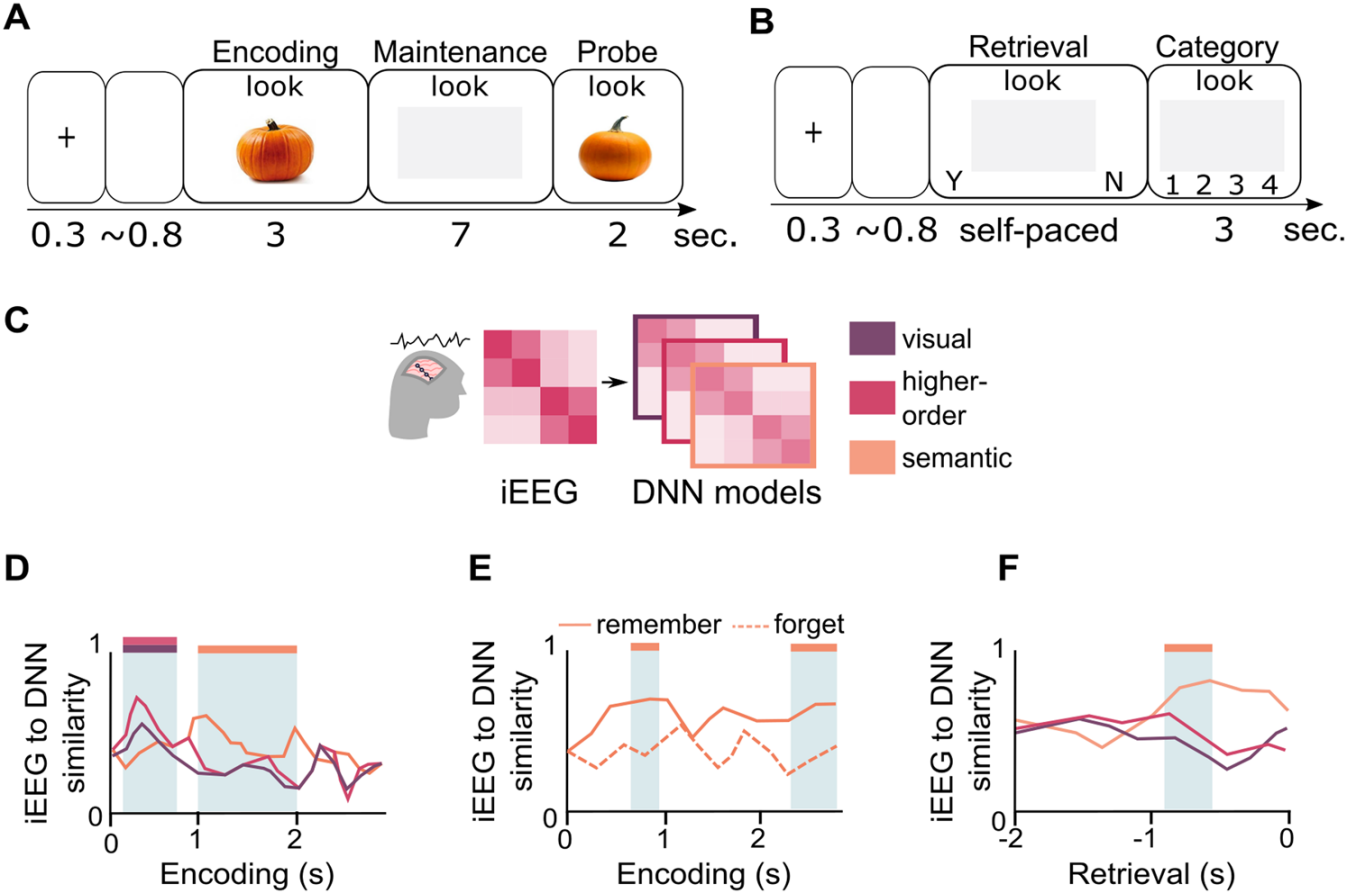
Representational formats during visual short-term memory maintenance and long-term memory encoding and retrieval. **A** Participants saw cue-object pairs and maintained the objects during a long maintenance period, which was followed either by a picture of the same item or of a similar lure. **B** During a subsequent long-term memory test, the cue word was presented, and participants were asked to vividly imagine the associated image. Afterwards they conducted a forced choice test on the category of the image. **C** Analysis methods: We combined RDMs from eight different layers of a cDNN and from a dNLP to model lower-order visual, higher-order visual and semantic representational formats, respectively. Model RDMs were then compared to corresponding RDMs from intracranial EEG data during the different task periods. **D** During encoding, higher-order visual formats were gradually transformed into semantic representational formats. **E** More pronounced semantic formats during encoding predicted subsequent long-term memory success. **F** Semantic but not visual formats were found during successful memory retrieval (s=seconds)

A very recent study thus set out to investigate if DNN similarities would reflect neural similarities during a visual short-term memory (VSTM) task (Fig. 2A), with VSTM being the earliest offline processing stage following perception (Liu et al. 2020). A follow-up study (Fig. 2B) then tested the effects of short-term maintenance and consecutive transformation stages on LTM retrieval (Liu et al. 2021) to test whether semantization already occurs during VSTM and whether early semantization may improve LTM performance.

VSTM is defined as the active maintenance of visual information for a short period of time in a limited capacity store (Baddeley and Hitch 1974; Luck and Vogel 1997). Current research suggests an important role of VSTM for integrating information, bridging the gap between perception and long-term memory (Chota and Van der Stigchel 2021), specifically involving regions along the VVS (Meyers et al. 2008; Cichy et al. 2014). Studies indicate “dynamic coding” with neurons carrying different information across the maintenance period (Stokes 2015), reflected by distinct representational formats. Along the VVS, these distinct formats have already been observed during object recognition (e.g., Devereux et al. 2018). Is there evidence for different representational formats present already during VSTM and for a shift from perceptual to semantic formats prior to LTM retrieval?

To address this question, we first analyzed similarities of neural patterns during an encoding and a maintenance period in a delayed matching to sample task (Fig. 2A) while participants (presurgical epilepsy patients) underwent intracranial EEG (iEEG) recordings (Liu et al. 2020). We then examined whether neural patterns during encoding reappeared during maintenance and long-term memory retrieval (Liu et al. 2021). During the maintenance period of the VSTM task, we found item-specific reinstatement of information from two distinct time windows during encoding, an early (250–770 ms post stimulus onset) and a later period (1000–1980 ms post stimulus onset), suggesting that both periods may contain different representational formats. Further analyses revealed higher item-specificity for the late encoding time window, indicating that specifically late representational formats are maintained faithfully during VSTM. Thus, neural similarity analysis revealed reinstatement of two distinct formats, but how exactly can these formats be characterized?

The integration of visual input with long-term knowledge suggests an involvement of semantic information (Cichy et al. 2014; Stokes 2015), while cDNNs from object recognition capture visual and higher-order visual features only. Thus, we decided to combine a cDNN with a dNLP model to investigate matching of neural representational formats to either visual formats from the cDNN or semantic formats from the dNLP model (Fig. 2C).

Current theories suggest an involvement of VSTM in the transformation of visual stimuli into abstract long-term memory representations (Meyers et al. 2008; Cichy et al. 2014; Stokes 2015). Accordingly, we found evidence for visual features during stimulus encoding periods followed by abstract semantic representations during later processing periods, indicating a transformation of representational formats from sensory to abstract (i.e., non-perceptual) formats (Fig. 2D). Specifically, the absence of sensory information during the later period suggests an interplay between bottom-up visual processing during the early and semantic top-down processing during later processing steps—possibly reflecting the integration of novel sensory stimuli into long-term memory stores (Clarke 2015; Jozwik et al. 2017; O’Donnell et al. 2018). In addition, the presence of a semantic format may be beneficial in order to transform stimuli into a lower-dimension representation with reduced information content, which may provide a functional benefit: Conci et al. (2021) found VSTM capacity to be linked to participants’ prior knowledge, with higher capacity if the stimulus meaning was known.

Recent findings from studies using fMRI provide additional evidence of shared representational formats between VSTM and long-term memory retrieval (Vo et al. 2022). Bainbridge et al. (2021) found different levels of abstraction when comparing encoding and retrieval representations. Whereas both fine-grained (e.g., penguin, lion) and coarse (e.g., bird, feline) features were observed during encoding, primarily coarse features were present during recall. Specifically, their results demonstrate a shift from the VVS showing peak activity during encoding to anterior areas during retrieval. Yet, this study does not show a complete loss of perceptual (e.g., fine-grained) information that would reflect a transformation of the same memory trace since this perceptual information was still observable in some areas. Whereas Audrain and McAndrews (2022) also found that memory representations became coarser over time, interestingly they found this generalization was linked to prior knowledge with only congruent semantic stimuli associations integrated in the medial prefrontal cortex (mPFC). This suggests that rapid semantization, i.e., due to congruency to prior knowledge, can facilitate memory transformation.

A follow-up analysis on the results during VSTM described above supports these findings even further (Liu et al. 2021): In line with our results from the maintenance period, we found that transformation into semantic formats was linked to subsequent LTM performance. Specifically, remembered images showed more pronounced semantic formats during encoding compared to forgotten images (Fig. 2E) and were linked to the occurrence of semantic but not sensory formats during retrieval (Fig. 2F). Interestingly, item-specific memory representations during retrieval were more similar to the visual short-term maintenance period compared to encoding. Together with better memory performance when conceptual formats were abstracted during encoding, the findings of these studies suggest semantization already happening at early stages of memory (i.e., encoding and VSTM) which in turn leads to better long-term memory formation. Overall, there seem to be parallel generalization processes during both encoding and consolidation, modulated by factors such as prior knowledge, which are fundamental to memory formation in both humans and neural networks (Kumaran et al. 2016) and supposedly are not limited to post-encoding consolidation periods.

## Beyond sensory formats—affective and contextual dimensions

In previous sections we focused on perceptual and conceptual formats, yet we hardly believe that these two cover the entirety of representational dimensions in neurocognitive processing (Gilboa and Moscovitch 2021). There might be additional, more abstract formats (e.g., involving scripts and schemata) or additional dimensions, such as the affective evaluation or the contextual embedding of an episode. When we think back to the example described above on a day at the beach, we may not only remember its multisensory aspects (e.g., the feeling of the sand, the sound of waves) but also the emotions we felt in that moment.

Indeed, there is evidence for neural representations of affective dimensions and categories across large-scale brain networks (Kragel and LaBar 2016), even spanning to areas along the VVS (Kragel et al. 2019). Concerning memory representations of emotional contents and their potential contextual embedding, emotions have been shown to modulate memory formation via processes of emotional binding (Mather 2007; Yonelinas and Ritchey 2015). Typically, emotions enhance memory (Talmi 2013; LaBar and Cabeza 2006), and this is particularly the case for negative emotions (Kensinger 2007). Interestingly, negative emotions seem to specifically enhance certain representational formats, with some studies indicating better accessibility of perceptual formats (Kensinger et al. 2006, 2007). Similar effects may occur for negative emotions induced by psychosocial stress, a particularly ecologically relevant condition (Freund et al. 2023). How will affective evaluation and contextual embedding affect neural representations of different stimuli of a stressful episode?

It is well established that the effects of stress on memory depend on the phase (Roozendaal 2002; Het et al. 2005; Joëls et al. 2006; Wolf 2009; Shields et al. 2017) of memory processing. While stress before or during encoding may have mixed effects, it is usually beneficial when experienced after encoding or during consolidation. By contrast, experiencing stress shortly before or during retrieval is consistently detrimental to performance (de Quervain et al. 1998; Wolf 2009; Shields et al. 2017).

**Fig. 3:**
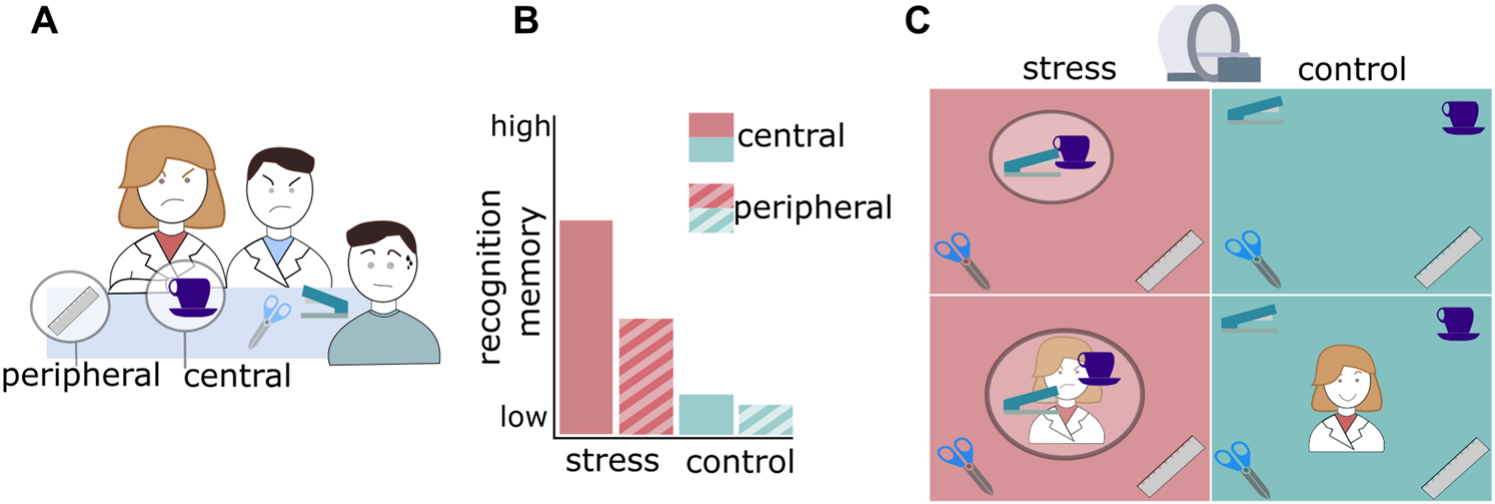
Effects of psychosocial stress on memory representations. **A** Participants conducted a psychosocial stress intervention (Trier Social Stress Test, TSST) in which some objects were manipulated by stress-inducing committee members (central objects) while others were not (peripheral objects). A second group of participants took part in a non-stressful control version of the task. **B** We found that central objects were better remembered than peripheral objects, and that this effect was enhanced in stressed participants. **C** On the next day, pictures of all objects were presented in the MRI scanner, and we measured their representational similarity. In the left amygdala, we found that central objects were more similar to other objects of the same episode and dissimilar to distractor objects when comparing stressed vs. control participants (upper row). Better memory performance for these objects was explained by the similarity of their representations to the representation of the stressor, i.e., the committee members’ faces (lower row)

In order to investigate memories of a stressful episode, we applied an ecologically valid experimental design in which stimuli are incidentally encoded during a psychosocial stress intervention (Wolf 2019). In the Trier Social Stress Test (TSST; Kirschbaum et al. 1993), participants conduct a mock job interview in front of a neutrally acting committee. In previous studies, Wiemers et al. adapted the TSST to contain a number of different everyday objects (Wiemers et al. 2013; Wolf 2019). In this version of the TSST (Fig. 3A), the interview room and especially the table in front of the committee are equipped with a number of different objects, which are incidentally encoded during the TSST. Half of these objects are manipulated by the committee members in a standardized way to render them more salient for the participant (“central objects”), while other objects are not manipulated (“peripheral objects”). Several studies showed that central objects are better remembered than peripheral objects, and that this effect is increased by psychosocial stress (Wiemers et al. 2013, 2014; Herten et al. 2017a, b). These results are in line with the hypothesis that stress particularly enhances the encoding of central cues (Easterbrook 1959; Wolf 2019).

We speculated that stress may enhance later memory for central objects by supporting generalization or binding processes of their neural representations. The term “binding” typically refers to the formation of integrated representations of multiple aspects of an episode, i.e., of different elements within one spatiotemporal context, and has been proposed to rely critically on the hippocampus (Ranganath 2010; Eichenbaum 2017). Yonelinas and Ritchey (2015) have suggested an “emotional binding” account according to which an emotion instead of the spatio-temporal context binds the features of an episode. They proposed that emotional binding occurs in the amygdala, that it may outweigh spatio-temporal binding processes in the hippocampus, and that this is the reason why emotional memories are less likely to be forgotten. Another binding approach proposed by Mather (2007) may provide an explanation for the superior memory of the central aspects in an emotional episode. In her “object-based framework”, she suggests that emotionally arousing objects attract attention and that this is the reason why the constituent features of the object are bound and well-remembered.

On the neural level, these binding approaches would predict higher similarity (lower representational distances) between neural representations of central objects. Generalization effects in humans have been previously found in a fear learning paradigm and were predictive of long-term fear memories (Visser et al. 2011, 2013). Specifically, pattern similarity changes in ventromedial PFC at the time of learning could predict the behavioral expression of long-term fear learning, i.e., changes in pupil dilation (Visser et al. 2013). In addition, fear learning led to generalization in other brain regions such as anterior cingulate cortex, amygdala, and superior frontal gyrus (Visser et al. 2011). These results suggest that increased pattern similarity between conditioned and unconditioned stimuli supports fear conditioning in a variety of brain regions including the amygdala—i.e., that higher pattern similarity in these regions reflects generalization and binding processes.

We investigated the effects of stress on memory representations and their impact on subsequent recognition memory (Bierbrauer et al. 2021). We conducted the TSST (Fig. 3A) and a non-stressful control version and tested memory performance for central and peripheral objects and the faces of the committee members. In line with previous studies (Wiemers et al. 2013), central objects were generally better remembered than peripheral objects (Fig. 3B). This effect was significantly more pronounced for stressed participants. Using fMRI, we measured the neural representations of central and peripheral objects and of the faces (Fig. 3C). Interestingly, we found that neural representations of central objects in the stressful episode became more similar to other objects from the same episode and dissimilar to distractor objects (i.e., objects that belonged to other potential episodes). In addition, we could explain higher memory performance for these objects by the similarity of their representations to the representation of the stressor, i.e., the committee members’ faces. This suggests that the beneficial effects of stress on memory formation rely on a generalization of neural representations within the stressful episode, which is driven by higher similarity with the representation of the stressor. This representational change may also explain why memories of stressful experiences can be triggered by neutral cues with low representational distance to the stressor.

Our study demonstrates that investigating the representational structure or “geometry” of affective and contextual dimensions of memory traces may provide mechanistic insights into representational formats beyond perceptual and conceptual dimensions. In other words, understanding how neural representations are transformed by factors such as stress will help us understand how these factors change our memories. In the future, it would be interesting to link perceptual and conceptual format from DNNs to data from affective episodes to further broaden the understanding of how affective evaluation and contextual embedding modulates these formats, and how they may act as additional formats.

## Conclusions

We started out describing several aspects of the memory for a recently experienced episode. The mental “image” of this episode as well as its non-sensory aspects relate to neural representations in various brain regions, across several levels of brain organization and in different representational formats. We described that RSA is well suited to assess the structure of memory representations (i.e., their representational geometry) and that we can employ DNNs to differentiate multiple representational formats. Not only can we quantify the formats themselves, but we also gain insights into how one format is transformed into another format and how this process may benefit memory consolidation and long-term memory retrieval. Importantly, we can demonstrate that generalization is not limited to consolidation but may also happen more rapidly, i.e., during encoding and maintenance. In this review we highlighted visual, higher-order visual and semantic formats that can be easily modeled by current cDNN and dNLP architectures. These models only provide a first approximation to the large variety of representational formats that are processed in the brain, including formats along dimensions such as affective evaluation or contextual embedding. In addition, they indicate the importance of combining computational and neuroscientific methods to understand memory. We propose that elucidating the neural representations underlying episodic memories should be a major goal in memory research.

## Data Availability

Enquiries about data availability of the experiments presented in this review should be directed to the corresponding author.
